# Neighbor Relatedness Contributes to Improvement in Grain Yields in Rice Cultivar Mixtures

**DOI:** 10.3390/plants14152385

**Published:** 2025-08-02

**Authors:** You Xu, Qin-Hang Han, Shuai-Shuai Xie, Chui-Hua Kong

**Affiliations:** 1 College of Resources and Environmental Sciences, China Agricultural University, Beijing 100193, China; xuyou406@126.com (Y.X.); hqhcau@163.com (Q.-H.H.); xie18434056985@126.com (S.-S.X.); 2 Institute of Ecological Conservation and Restoration, Chinese Academy of Forestry, Beijing 100091, China

**Keywords:** effect of relatedness, flowering time, kin recognition, neighboring cultivar identity, nitrogen use level, rhizosphere microbial community

## Abstract

The improvement in yield in cultivar mixtures has been well established. Despite increasing knowledge of the improvement involving within-species diversification and resource use efficiency, little is known about the benefits arising from relatedness-mediated intraspecific interactions in cultivar mixtures. This study used a relatedness gradient of rice cultivars to test whether neighbor relatedness contributes to improvements in grain yields in cultivar mixtures. We experimentally demonstrated the grain yield of rice cultivar mixtures with varying genetic relatedness under both field and controlled conditions. As a result, a closely related cultivar mixture had increased grain yield compared to monoculture and distantly related mixtures by optimizing the root-to-shoot ratio and accelerating flowering. The benefits over monoculture were most pronounced when compared to the significant yield reductions observed in distantly related mixtures. The relatedness-mediated improvement in yields depended on soil volume and nitrogen use level, with effects attenuating under larger soil volumes or nitrogen deficiency. Furthermore, neighbor relatedness enhanced the richness and diversity of both bacterial and fungal communities in the rhizosphere soil, leading to a significant restructuring of the microbial community composition. These findings suggest that neighbor relatedness may improve the grain yield of rice cultivar mixtures. Beneficial plant–plant interactions may be generated by manipulating cultivar kinship within a crop species. A thorough understanding of kinship strategies in cultivar mixtures offers promising prospects for increasing crop production.

## 1. Introduction

Mixing crop cultivars has been recognized as an ecological and evolutionary strategy to control crop diseases and pests, subsequently increasing crop yields, and it is experiencing a revival of interest in agricultural practices [1,2,3]. Cultivar mixtures improve plant production and temporal yield stability. The improvement can be attributed to genetic diversity, resource use efficiency, and the establishment of a beneficial soil microbial community [4,5,6]. Furthermore, the success of cultivar mixtures heavily depends on the neighboring cultivar identity, particularly due to neighbor-identity-specific changes in intraspecific plant–plant competition and nutrient uptake [7,8]. Successful cultivar mixtures benefit from reducing intraspecific competition and enhancing conspecific cooperation.

Genetic manipulation of crop phenotypes to reduce competitive ability can enhance plant production and increase yield [9]. However, the neighbor-dependent benefits of inter-varietal interactions among plants in genotypically diverse mixtures have rarely been explored in the context of relatedness-mediated plant–plant interactions [10]. Recent studies have shown that plants can distinguish their conspecific neighbors based on their relatedness, enabling them to optimize response strategies to the composition of their local neighborhood [11,12,13]. In particular, plants plastically adjust their floral and clonal allocation in response to their neighbor’ identity via kin discrimination [14,15]. Accordingly, neighbor relatedness can reduce intraspecific competition and alter local conditions, improving group performance and fitness [16,17,18]. Importantly, the ability to discriminate in favor of relatedness drives conspecific cooperation toward closely related combinations, promoting the survival and reproduction of relatives [19,20]. Such a relatedness-mediated conspecific cooperation within a species occurs at the individual, population/biotype [12,21,22], and cultivar levels [23,24] in both natural and cropping systems. However, the association of soil microbes has not been explored in the context of neighbor relatedness recognition and response.

Relatedness-mediated conspecific cooperation in crop plants can result in less intraspecific competition, maximizing stand performance and increasing yield [25,26]. The effect of relatedness has been observed in several crop species, such as rice (*Oryza sativa* L.) [27], maize (*Zea mays* L.) [28], soybean (*Glycine max* (L.) Merrill) [13], broomcorn (*Sorghum vulgare* var. *technicum* [Korn.]) [29], and pea (*Pisum sativum* L.) [24]. Preferentially enhancing conspecific cooperation and reducing neighbor competitive effects on relatives may be a potential mechanism responsible for the increased yield of crop cultivar mixtures [10,23,30]. However, most studies on relatedness-mediated conspecific cooperation in crop species have focused on early vegetative growth and biomass allocation under controlled conditions, with limited attention on field yield with nutrient use efficiency and soil microbial communities. A lack of knowledge about reproductive traits and grain yield in field situations calls for further realistic studies.

Rice is a principal grain crop comprising numerous cultivars with varying degrees of genetic relatedness. Breeding generates rice cultivars that are genetically and morphologically uniform and uses fixed lines, with no genetic variation within a given cultivar. Therefore, self-pollinated rice is a better model for studying relatedness-mediated conspecific cooperation at the cultivar level compared to outcrossing species where genetic variation within cultivars is higher [23,31,32]. Improvement in grain yields has been observed in mixtures of closely related rice cultivars under field situations and controlled conditions [18,23,27,30]. However, the effects of relatedness on grain yield across site-years remain fragmentary. In this study, a comparative approach involving one focal cultivar grown with three different neighbors was employed to investigate the effects of relatedness on grain yields in rice cultivar mixtures across site-years. Furthermore, it was hypothesized that neighbor relatedness may modify soil microbial communities, resulting in altered biomass allocation, flowering time, and grain yield. To achieve this, a series of field and greenhouse experiments were carried out with a relatedness gradient of rice cultivars, varying plant arrangements, growing years, and nitrogen use levels. These experiments were used to (1) assess relatedness-mediated impacts on rice biomass allocation, flowering time and grain yield, (2) examine how nutrient use efficiency drives these changes, and (3) determine changes in the rhizosphere soil microbial community. These efforts thus provide compelling evidence of an outstanding phenomenon: neighbor relatedness for improving grain yields in rice cultivar mixtures.

## 2. Results

### 2.1. Effect of Neighbor Relatedness on Grain Yields

**Figure 1:**
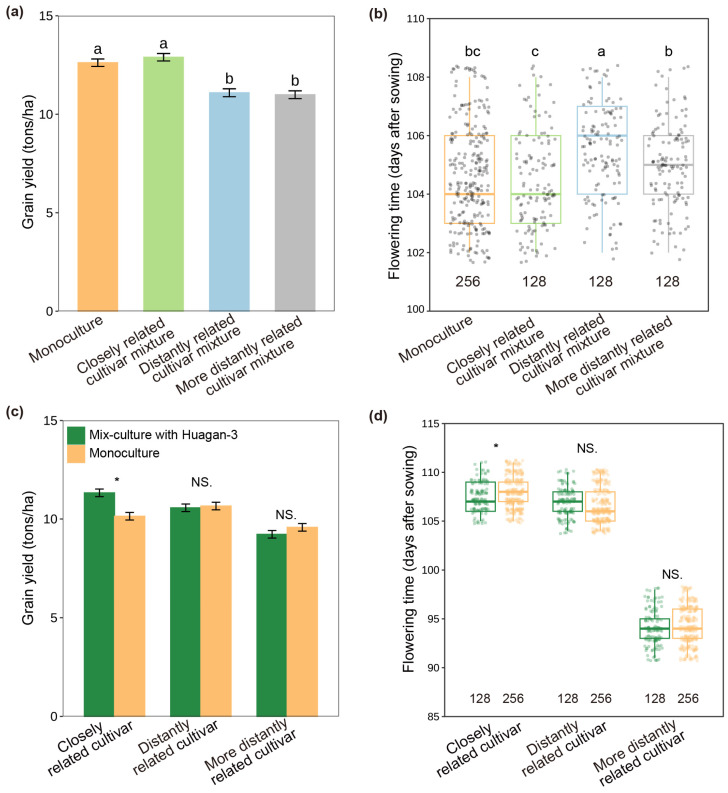
Effect of neighbor kinship on rice flowering and grain yield in paddy fields with cross pattern in 2021. (**a**) Grain yield of focal cultivar (Huagan-3) in monoculture and mix culture with closely and distantly related cultivars. (**b**) Flowering time of focal cultivar in monoculture and mix culture with closely and distantly related cultivars. (**c**) Grain yields of closely and distantly related cultivars in monoculture and mix culture with focal cultivar. (**d**) Flowering times of closely and distantly related cultivars in monoculture and mix culture with focal cultivar. Closely related cultivar, Huagan-8; distantly related cultivar, Huafeng (indica); more distantly related cultivar, Liaojing-9 (japonica). Values below the boxes are the numbers of rice samples for flowering time. Values plotted are means ± SE. Columns with the same letter among cultivar mixtures are not significantly different at *p* < 0.05 by Tukey HSD tests. NS, no significant differences. Asterisks indicate significant differences between monoculture and mix culture in each cultivar using Student’s *t*-test (* < 0.05).

When rice cultivars were grown together and interacted in paddy fields, their flowering time and grain yields varied significantly with interacting cultivars and their relatedness (Figure 1; F = 24.340, *p* < 0.001). The focal cultivar (Huagan-3) growing with the closely related cultivar (Huagan-8) had significantly earlier flowering and increased grain yield compared to those growing with distantly related cultivars (Huafeng and Liaojing-9) (Figure 1a,b). Interestingly, the flowering time and grain yield of the closely related cultivar (Huagan-8) were similarly facilitated by the focal cultivar (Huagan-3) compared to the monoculture, but a similar facilitation was not observed by distantly related cultivars (Huafeng and Liaojing-9) (Figure 1c,d). The results clearly indicated that beneficial plant–neighbor interaction occurred in the closely related cultivar mixture.

**Figure 2:**
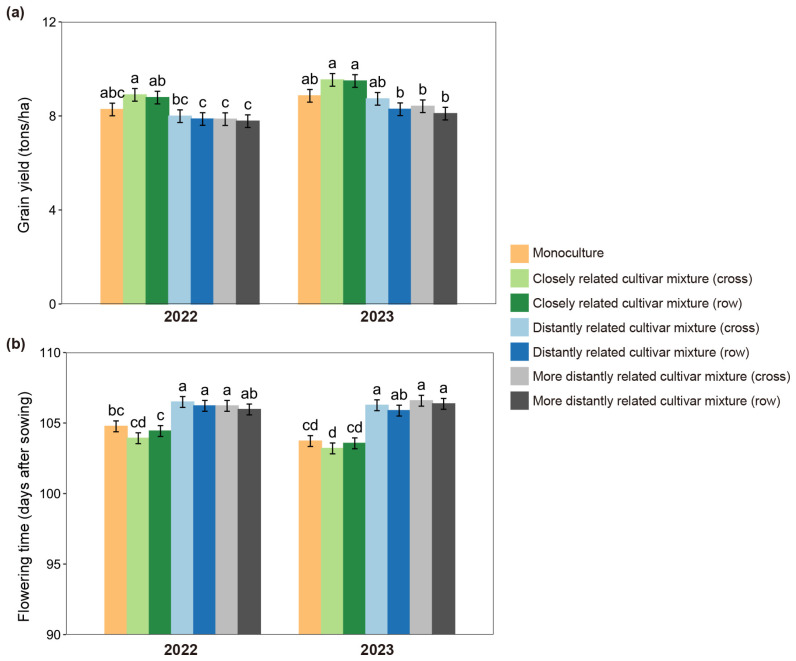
Grain yields (**a**) and flowering times (**b**) of focal cultivar (Huagan-3)-based monoculture and mixtures of closely and distantly related cultivars across different planting patterns (cross vs. row) in two years (2022 vs. 2023). Closely related cultivar, Huagan-8; distantly related cultivar, Huafeng (indica); more distantly related cultivar, Liaojing-9 (japonica). Values plotted are means ± SE. Columns with the same letter are not significantly different in terms of relatedness and planting pattern at *p* < 0.05 by Tukey’s HSD tests.

Significant effects of relatedness were further observed in root-to-shoot ratio, flowering time, and grain yield in both 2022 and 2023 (Figure 2 and Figure S1; Table S1). When grown with the closely related cultivar, a significantly lower root-to-shoot ratio occurred, whether plants were at seedling or mature stages (Figure S1). Compared with monoculture, grain yield increased over 5% (Figure 2a) and flowering time advanced 1–3 days (Figure 2b) in the closely related cultivar mixture. However, similar changes in grain yield and flowering time were not observed in the focal cultivar grown with distantly related cultivars, particularly for the more distantly related cultivar (Figure 2a,b). Furthermore, no significant differences were observed between cross and row in root-to-shoot ratio, flowering time, or grain yield among closely related cultivar or distantly related cultivar mixtures, but significant differences occurred between 2022 and 2023, probably due to a large difference in rainfall (Figure S2; Table S1). However, neither the planting pattern nor year altered the effect of relatedness in the closely related cultivar mixture.

### 2.2. Soil Volume and Nitrogen Use Level Influence the Effect of Relatedness

**Figure 3:**
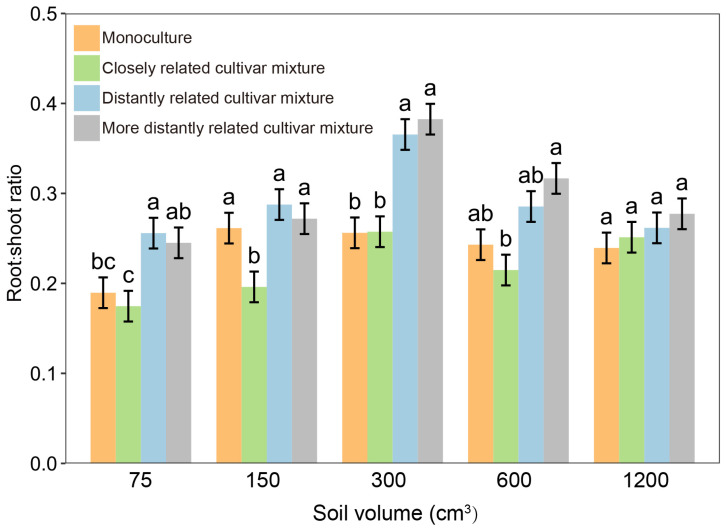
Root-to-shoot ratio of focal cultivar (Huagan-3) in monoculture and mixtures of closely and distantly related cultivars across different soil volumes. Closely related cultivar, Huagan-8; distantly related cultivar, Huafeng (indica); more distantly related cultivar, Liaojing-9 (japonica). Values plotted are means ± SE. Columns with the same letter are not significantly different in terms of relatedness at *p* < 0.05 according to one-way ANOVA, followed by Tukey’s HSD tests.

**Figure 4:**
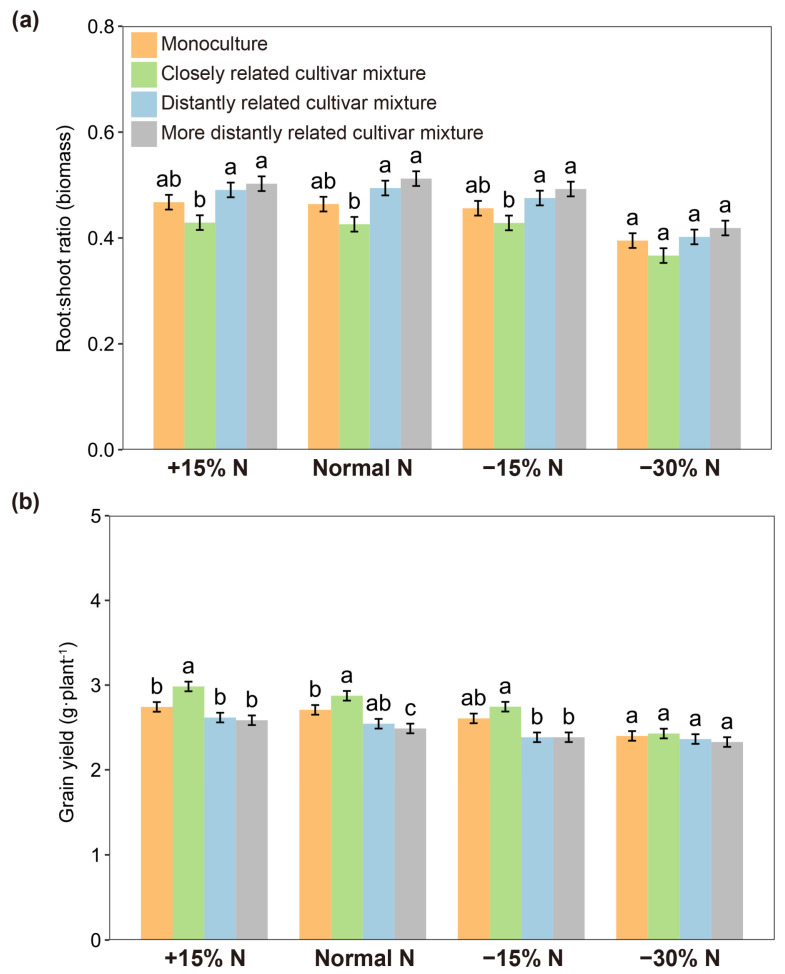
Root-to-shoot ratio (**a**) and grain yields (**b**) of focal cultivars (Huagan-3) in monoculture and mixtures of closely and distantly related cultivars at different nitrogen use levels. Closely related cultivar, Huagan-8; distantly related cultivar, Huafeng (indica); more distantly related cultivar, Liaojing-9 (japonica). Values plotted are means ± SE. Columns with the same letter are not significantly different in terms of relatedness at *p* < 0.05 followed by Tukey’s HSD tests.

When each focal cultivar was paired with closely and distantly related cultivars in pots, the root-to-shoot ratio of rice plants varied not only with the relatedness of paired cultivars but also their growing soil volume (Figure 3). Within soil volumes of 75–600 cm^3^, the root-to-shoot ratio of closely related cultivar mixtures was significantly lower than that of distantly related cultivar mixtures, whereas there was no significant difference at a 1200 cm^3^ soil volume (Figure 3; Table S2), indicating that the effect of relatedness in the closely related cultivar mixture could be attenuated by a larger soil volume. Similar results were observed for nitrogen use levels. Under normal nitrogen, 15% excess nitrogen, and 15% reduced nitrogen conditions, the root-to-shoot ratio of closely related cultivar mixtures was significantly lower than that of distantly related cultivar mixtures, but a significant difference was not observed at a 30% nitrogen reduction (Figure 4a; Table S3). Furthermore, the grain yield of closely related cultivar mixtures was significantly higher than that of distantly related cultivar mixtures when normal nitrogen, 15% excess nitrogen, and 15% reduced nitrogen were applied. However, when the nitrogen use level was reduced by 30%, the yield increase was no longer significant among cultivar mixtures regardless of genetic relatedness (Figure 4b).

### 2.3. Effect of Neighbor Relatedness on Rhizosphere Soil Microbes

**Figure 5:**
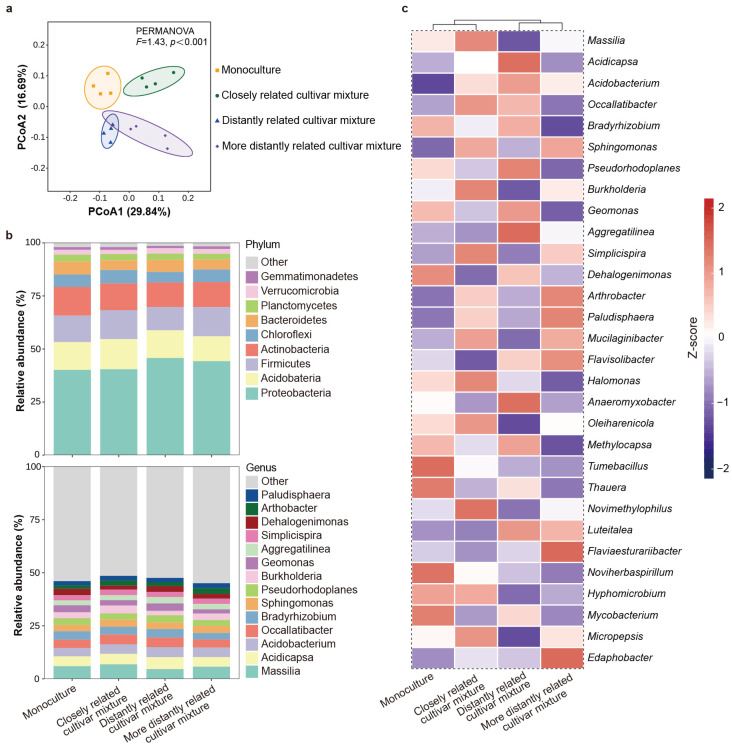
Rhizosphere soil bacterial community of focal rice cultivar (Huagan-3) in monoculture and mixtures of closely and distantly related cultivars. Closely related cultivar, Huagan-8; distantly related cultivar, Huafeng (indica); more distantly related cultivar, Liaojing-9 (japonica). (**a**) Principal coordinate analysis (PCoA) ordination of bacterial community structures in rhizosphere soil based on Bray–Curtis distance. Ellipse is drawn assuming multivariate normal distribution (confidence level: 0.85). (**b**) Taxonomic composition of bacterial community in rhizosphere soil at phylum and genus level. For bacteria, only relative abundances over 1% are shown. (**c**) Comparison of bacterial average relative abundance at genus level in rhizosphere soil. Relative abundance of each genus is colored according to row z score (value − row mean)/row standard deviation); only top 30 relative abundances are shown.

The α-diversity of bacteria in the rhizosphere soil of the focal cultivar varied with the relatedness of mixing neighbors. Compared with the mixture of the focal cultivar with itself or distantly related cultivars, a significantly higher richness and Shannon index were observed in the mixture of the focal cultivar with the closely related cultivar, but there was no significant difference in the Chao 1 index (Table S4). The results of β-diversity analysis showed that the rhizosphere soil bacterial community composition of the focal cultivar paired with the closely related cultivar were distinct from those found for the focal cultivar paired with itself or with the distantly related cultivar, which clustered together, and the distantly related cultivar and the more distantly related cultivar were clustered together (Figure 5a). Furthermore, relative abundance of bacteria phyla exhibited that *Proteobacteria* (42.66%), *Acidobacteria* (13.00%), *Firmicutes* (12.72%), *Actinobacteria* (12.36%), *Chloroflexi* (5.77%), and *Bacteroidetes* (5.26%) dominated the soil microbiome across all groups. At the genus level, *Massilia* (5.78%), *Acidicapsa* (4.94%), *Acidobacterium* (4.38%), *Occallatibacter* (4.21%), and *Bradyrhizobium* (3.72%) were the dominant bacterial taxa across all soil samples (Figure 5b). Specifically, the relative abundances of *Massilia, Burkholderia, Halomonas, Novimethylophilus*, and *Micropepsis* were higher, while those of *Dehalogenimonas* and *Flavobacterium* were lower in the rhizosphere soil of the focal cultivar mixed with closely related cultivars compared to the focal cultivar mixed with itself or with distantly related cultivars (Figure 5c).

**Figure 6:**
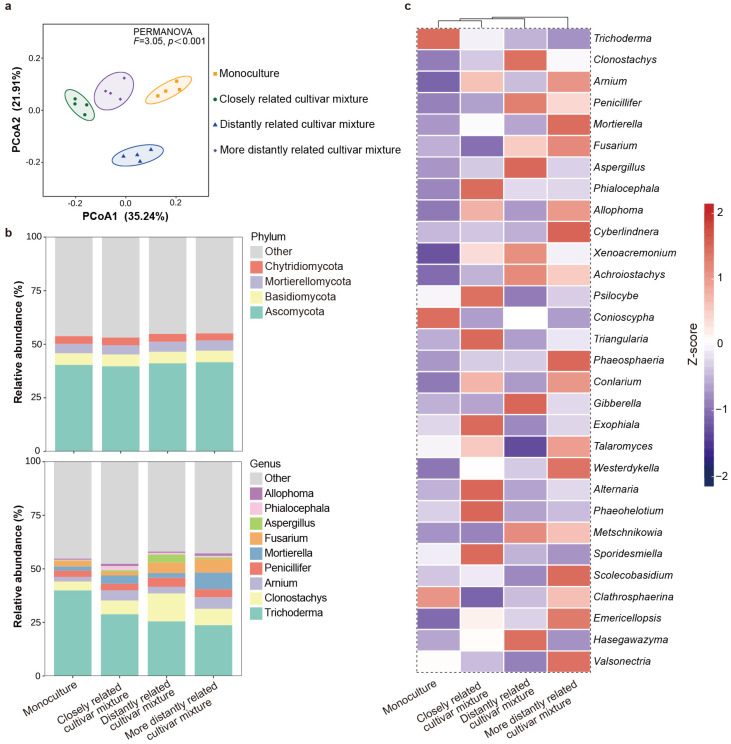
Rhizosphere soil fungal community of focal rice cultivar (Huagan-3) in monoculture and mixtures of closely and distantly related cultivars. Closely related cultivar, Huagan-8; distantly related cultivar, Huafeng (indica); more distantly related cultivar, Liaojing-9 (japonica). (**a**) Principal coordinate analysis (PCoA) ordination of fungal community structures in rhizosphere soil based on Bray–Curtis distance. Ellipse is drawn assuming multivariate normal distribution (confidence level: 0.85). (**b**) Taxonomic composition of fungi community in the rhizosphere soil at phylum and genus level. For fungi, only relative abundances of over 1% are shown. (**c**) Comparison of fungal average relative abundance at genus level in rhizosphere soil. Relative abundance of each genus is colored according to row z score ((value − row mean)/row standard deviation); only top 30 relative abundances are shown.

For fungal α-diversity, the focal cultivar paired with the closely related cultivar showed higher observed species richness and Shannon indices compared to the focal cultivar paired with distantly related cultivars. However, no significant difference in the Chao 1 indices was detected among the mixtures (Table S4). With regard to β-diversity, fungal communities were significantly different from each other in all the four compartments (Figure 6a). In all rhizosphere soils, the dominant phyla were *Ascomycota* (40.68%), *Basidiomycota* (5.40%), *Mortierellomycota* (4.54%), and *Chytridiomycota* (3.55%); the dominant genera were *Trichoderma* (29.48%), *Clonostachys* (7.81%), *Arnium* (3.84%), *Penicillifer* (3.93%), *Mortierella* (3.90%), and *Fusarium* (3.50%) (Figure 6b). Specifically, *Phialocephala, Psilocybe, Triangularia, Exophiala, Alteraria, Phaeohelotium,* and *Sporidesmiella* were more abundant, while *Fusarium* and *Clathrosphaerina* were less enriched in the rhizosphere soil of the focal cultivar mixed with the closely related cultivar compared to other cultivars (Figure 6c).

## 3. Discussion

The importance of relatedness-mediated intraspecific plant–plant interactions and their consequences in natural and managed ecosystems have received a great of attention in the past decade [33,34,35]. Recent efforts have made considerable progress in understanding how neighbor relatedness can improve grain yield in crop cultivar mixtures [10,23,26,30]. Using an established relatedness gradient of rice cultivar mixtures in paddies, we found that the grain yield of closely related cultivar mixtures was consistently greater than that of distantly related cultivar mixtures. The benefit was generated by a reduced root-to-shoot ratio and accelerated flowering in closely related cultivars regardless of plant patterns. In particular, the effect of relatedness was accompanied by an alteration in the rhizosphere soil microbial community. In contrast, distantly related cultivars exhibited stronger competitive behaviors, such as increased root allocation and delayed flowering, which likely reduced resource use efficiency and ultimately limited grain yields. While grain yield differences between closely related mixtures and monoculture were not statistically significant, the significant yield reduction in distantly related mixtures suggests that competitive costs from distant neighbors, rather than benefits from close kin, primarily drive the observed patterns. The differential responses to neighbor relatedness suggest that rice cultivars may distinguish between kin and non-kin neighbors, though the significant yield reductions in distantly related mixtures appear more consequential than any potential kin benefits.

Improved grain yields can occur in closely related cultivar mixtures in paddies. This outstanding phenomenon has been evidenced in both genotypes of indica-inbred and indica-hybrid lines, with two sets of rice cultivar mixtures at different levels of genetic relatedness in recent years [23,30]. The current study documents this phenomenon on another site in continuous years, showcasing the effects of relatedness in closely related cultivar mixtures on grain yield across site-years. Rice cultivars with kin recognition ability are a key for improved grain yields in closely related cultivar mixtures. The selection of the Huagan-3 set is justified because it previously showed an ability to adjust its performance to the relatedness of neighboring cultivars [23,30]. However, the extent to which this kin recognition ability is generalizable in rice cultivars remains unclear and requires further investigation.

In natural systems, proliferative and intrusive root behaviors are likely essential for increasing access to soil resources. However, proliferative and intrusive root behaviors may be disadvantageous in cropping systems with plants allocating more biomass to root systems, reducing yields [36,37,38]. Kin recognition with conspecific cooperation among crop plants is predicted to increase yield by reducing competitive root systems and shifting resource allocation to reproduction. Neighbor relatedness can result in differences in growth and biomass allocation, particularly in root growth and placement [18,39,40]. In the current study, closely related rice cultivar mixtures with low root-to-shoot ratios reduced root allocation. Reducing the energy devoted to competitive root systems allows for greater allocation to reproduction, advancing flowering and increasing grain yield. Therefore, minimizing root–root competition through kinship strategies in cultivar mixtures is an ecological approach to creating more productive and resilient agroecosystems.

The differential responses to neighbor relatedness in plants may be modulated by environmental conditions, particularly for root spatial patterns and soil nutrient availability [19,27,41]. Soil volume is a key determinant for root partitioning and acquisition of nutrients in plant–plant interactions [37,39]. The data generated in this study demonstrated that soil volume and nitrogen use level influence the effects of relatedness in closely related cultivar mixtures to a certain extent. The effect of relatedness on rice cultivar mixtures was attenuated with larger soil volumes. This suggests that kinship strategies may easily occur in rice cultivar mixtures when soil space is limited. Furthermore, soil nutrient availability acts as an alternative explanation for the modulation of the effect of relatedness in rice cultivar mixtures. The importance of soil nutrient partitioning and cycling has been investigated in relatedness-mediated intraspecific plant–plant interactions [18,29,42]. A recent study has indicated that quinoa plants always grow better with kin than non-kin regardless of soil nutrient conditions [43]. In this study, the effects of relatedness in closely related cultivar mixtures can be maintained at a 15% fluctuation of normal nitrogen use level, but nitrogen deficiency may diminish the effect. Notably, under nitrogen deficiency (−30% N), neither closely nor distantly related mixtures maintained significant yield differences, implying that extreme resource limitation neutralizes kinship-driven advantages and disadvantages alike. This likely reflects plants prioritizing individual growth and survival over neighbor interactions under nutrient limitation [44].

Kinship strategies in conspecific plants are expressed through reduced root allocation, increased nitrogen uptake, and enhanced soil N nitrification by enriching functional microbial groups [13]. Indeed, plant–plant interactions can shape soil microbial communities, and these communities are influenced by the neighboring plant context. In particular, neighboring plant identity greatly influences the recruitment of the rhizosphere microbiome in target plants [45]. However, relatedness-mediated intraspecific plant–plant interactions have rarely been invoked in soil microbial communities. The current study reveals that neighbor relatedness alters soil microbial responses in rice cultivar mixtures.

The composition and diversity of rhizosphere microbial communities are recognized as essential indicators of plant performance [46]. Several studies have suggested that plant kin recognition contributes to the rhizosphere microbiome by forming larger mycorrhizal networks or enriching plant growth-promoting bacteria, thereby enhancing the root system’s capacity to absorb nutrients and resist soil-borne pathogens [7,47]. While higher genetic diversity is typically associated with increased microbial diversity [48], our study demonstrated that neighbor relatedness among rice cultivars increased the richness and diversity of rice rhizosphere bacteria and fungi in mixture systems. This finding suggests that distinct microbial interaction mechanisms may operate at intraspecific versus interspecific levels. Furthermore, neighbor relatedness significantly reshaped microbial community composition, increasing the relative abundance of representative bacteria genera such as *Massilia*, *Burkholderia*, and *Halomonas*. These bacteria are associated with plant growth, flowering, and reproduction [49,50,51]. Additionally, mixing with closely related cultivar compared to distantly related cultivars reduced the relative abundance of pathogenic fungi, such as those in the Fusarium genus. This reduction may be explained by the enrichment of potential antagonistic microorganisms, which suppress the growth of pathogens [52]. Importantly, rice plants in closely related cultivar mixtures exhibited significantly earlier flowering compared to those in distantly related cultivar mixtures. Phenotypic selection consistently favors early-flowering plants, particularly in temperate regions, likely due to advantages in pollinator access and seasonal fitness benefits [53]. The timing of plant flowering may be determined by rhizosphere microorganisms with specific microbes [51,54]. However, this study did not show which functional microbial groups could serve as drivers of rice flowering. Furthermore, plants alter the physical, chemical, and biological properties of soils in ways that affect the performance of themselves and co-occurring plants. This study indicates a pathway of kinship changing soil microbial compositions, which in turn influences plant trait responses. However, mechanisms regarding the effects of close relatedness on grain yield involve soil properties and the relationship between grain yield and microbial properties. Further studies on the critical mechanisms behind the kinship strategy in rice cultivar mixtures are warranted.

## 4. Materials and Methods

### 4.1. Rice Cultivars and Their Relatedness

Four rice (*Oryza sativa* L.) cultivars were used in this study: Huagan-3, Huagan-8, Huafeng, and Liaojing-9. Huagan-3 and Huagan-8 were bred from the same maternal parent, PI312777, with five reciprocal cross-combinations among different paternal parents [55]. Huafeng and Liaojing-9 are two commercial cultivars in the local rice industry of China that do not have consanguinity ties with Huagan-3 and Huagan-8. Huagan-3, Huagan-8, and Huafeng are indica-inbred rice lines, while Liaojing-9 is a japonica-inbred cultivar. Their pedigree and genetic distance are shown in Figure S3. Huagan-3 has been shown to exhibit kin recognition at the cultivar level [18,23]. Accordingly, Huagan-3 served as the focal cultivar, with Huagan-8 and Huafeng representing the closely related and distantly related cultivars, while Liaojing-9 was used as a more distantly related cultivar, resulting in a relatedness gradient of rice cultivars (Figure S3).

### 4.2. Field Trials

Field experiments were conducted in paddies during the 2021, 2022, and 2023 growing seasons at Suzhou Rice Experimental Station, China Agricultural University, Jiangsu Province, China. The experimental station is located in the Taihu plain on the southern side of the Yangtze River (Figure S4a). Precipitation and daily temperature throughout the rice-growing season in 2022 and 2023 are shown in Figure S2. Meteorological data showed similar average daily temperatures of 26.36 and 26.04 °C during the rice growth period in 2022 and 2023, but large differences occurred in rainfall, with 474.90 mm in 2022 and 923.50 mm in 2023, and the rainfall in 2022 was 448.60 mm lower than that in 2023 (Figure S2). The paddy soil is characterized as a typical fluvaquent, Entisol (US taxonomy), with pH 5.41, an organic matter content of 24.38 g·kg^−1^, total nitrogen of 1.51 g·kg^−1^, available phosphorus of 27.64 mg·kg^−1^, and available potassium of 40.23 mg·kg^−1^. The paddies had a history of rice cultivation over multiple previous seasons.

The field was divided into a series of 4 × 4 m^2^ plots, arranged in a completely randomized design with eight replicates (Figure S4b,c). The plots were subjected to four treatments in a cross or row pattern at a 1:1 ratio: (1) monoculture of Huagan-3, Huagan-8, Huafeng, or Liaojing-9 (monoculture); (2) mixture of focal cultivar (Huagan-3) with Huagan-8 (closely related cultivar mixture); (3) mixture of focal cultivar with Huafeng (distantly related cultivar mixture); and (4) mixture of focal cultivar with Liaojing-9 (more distantly related cultivar mixture) (Figure S4d). Each plot was separated by trenches with 50 cm discard strips on each side at a density of 50 plants/m^2^ by direct seeding. All field management practices, including water management (maintaining shallow flooding during tillering and intermittent irrigation during grain filling), fertilization (basal application of 7.5 g N/m^2^, 9.0 g P_2_O_5_/m^2^, and 8.5 g K_2_O/m^2^), and pest control (targeting rice planthoppers and sheath blight), followed the guidelines set by the local rural administration for rice cultivation, http://www.szwz.gov.cn/ (accessed on 6 April 2021).

In 2021, a preliminary experiment was conducted to investigate the effect of focal cultivar and neighboring cultivar interactions. Rice growth in each plot was checked weekly before flowering. The number of days from sowing when the first flower appeared in the main panicle was recorded daily in 256 randomly selected plants for the focal cultivar and 128 plants for each neighbor cultivar during the flowering stage. Finally, the grain yields of each cultivar in monoculture and mixtures were recorded at the mature stage.

In the years 2022 and 2023, roots and shoots were sampled by randomly selecting 200 rice seedlings from four out of eight replications in each plot at the five-leaf stage. The samples were then dried to a constant weight for biomass measurement and root-to-shoot ratio calculation. During the flowering period, the number of days from sowing to the appearance of the first flower in the main panicle was recorded daily in 150 randomly selected plants, regardless of cultivar, in the central area of each remaining plot. Finally, the total of grain yields in monoculture and mixtures were each measured in plots at rice maturity.

### 4.3. Greenhouse Experiments

Two experiments were carried out in a greenhouse at temperatures ranging from 20 °C to 30 °C, with relative humidity levels kept between 65% and 90% (Figure S5a). The soil was collected randomly from the surface (0–10 cm) of the paddy field at the experimental station described above and subsequently air-dried and sieved (2 mm mesh) to remove plant tissues for the following pot culture experiments. Each experiment was carried out using a completely randomized design with four replicates. The germinated seeds for each cultivar were surface-sterilized with a 5% H_2_O_2_ solution, placed on moistened filter paper in Petri dishes, and germinated in an environmental chamber at 28 °C in complete darkness.

A volume-mediated experiment was run to investigate the impact of soil volume on the root-to-shoot ratio of the focal cultivar in the presence of closely or distantly related cultivars from 15 May to 20 July 2024 (Figure S5b). Five pots of different sizes with diameters × heights of 6 cm × 6 cm, 8 cm × 7 cm, 12 cm × 10 cm, 18 cm × 12 cm, and 20 cm × 14 cm were filled with soil volumes of 75 cm^3^, 150 cm^3^, 300 cm^3^, 600 cm^3^, and 1200 cm^3^, respectively. In each pot, three germinated seeds of the focal cultivar (Huagan-3) were evenly spaced in the center, while three germinated seeds of the closely or distantly related cultivar were planted in the surrounding area. All treatments were replicated four times, with a total of 200 pots placed in the greenhouse. The pots were watered daily, and their positions were randomized weekly to ensure uniform environmental conditions. At the five-leaf stage, plants in each pot were sampled, and each pot containing 600 cm^3^ of soil was additionally used for the collection of rhizosphere soil, as described below. Finally, the shoots and roots of the focal cultivars were oven-dried for biomass measurements.

A nitrogen gradient experiment was conducted to evaluate the performance of the focal rice cultivar (Huagan-3) in response to neighbor relatedness under different nitrogen use levels between 1 June and 10 October 2024 (Figure S5b). A series of 18 (diameter) × 12 cm (height) plastic pots containing 4 kg of soil were used in the experiment. The base fertilizer application consisted of 30 mg P·kg^−1^ (KH_2_PO_4_) and 90 mg K·kg^−1^ (KCl). A nitrogen concentration gradient was established using normal fertilization, with nitrogen added to the soil as ammonium nitrate, resulted in four nitrogen use levels: +15% N (69 mg N·kg^−1^), Normal N (60 mg N·kg^−1^), −15% N (51 mg N·kg^−1^), and −30% N (42 mg N·kg^−1^). Two germinated seeds of the focal cultivar (Huagan-3) and two germinated seeds of closely or distantly related cultivars were planted in each pot in a completely cross pattern. Each nitrogen use level had eight replicates, and a total of 128 pots were placed in the greenhouse. The pots were watered daily and their positions randomized weekly. The focal cultivar in half of the pots was sampled at the five-leaf stage, and its shoots and roots were oven-dried for biomass measurements. In the remaining half of the pots, grain yield was recorded at maturity.

### 4.4. Rhizosphere Soil Sampling

The collection of rhizosphere soil from the focal cultivar (Huagan-3) in the pot containing 600 cm^3^ of soil primarily followed the method described by Edward et al. [56], with minor modifications. The roots of the focal cultivar were carefully extracted from the soil, and the loosely adhering soil was removed. The roots were then placed in a 50 mL centrifuge tube containing 10 mmol/L phosphate-buffered saline (PBS) with a pH of 7.2–7.4. The centrifuge tube containing the roots was placed on a vortex mixer and vortexed at 3000× *g*. After vortexing, the roots were carefully removed from the tube using sterilized forceps. The centrifuge tube was then sealed and placed in a centrifuge, where it was centrifuged at 8000× *g* for 10 min. Following centrifugation, the supernatant of the tube was carefully decanted and the soil pellet at the bottom was considered the rhizosphere soil. The soil samples were stored at −80 °C for subsequent DNA extraction and Illumina sequencing.

### 4.5. DNA Extraction, PCR Amplification, and Sequencing

Total DNA from soil samples was extracted using the Fast DNA Spin Kit for Soil (MP Biomedicals, Santa Ana, CA, USA). The procedural steps were carried out according to the manufacturer’s protocol. The quantity and quality of the extracted total soil DNA were assessed by NanoDrop ND-1000 spectrophotometer (Thermo Fisher Scientific, Waltham, MA, USA). DNA for microbial community determination was diluted to 5 ng µL^−1^. The primers used for amplifying the bacterial *16S rRNA* gene region were 515F (5′-GTGCCAGCMGCCGCGGTAA-3′) and 806R (5′-GGACTACVSGGGTATCTAAT-3′). The primers used for amplifying the fungal ITS region were ITS1-1F-F (5′-CTTGGTCATTTAGAGGAAGTAA-3′) and ITS1-1F-R (5′-GCTGCGTTCTTCATCGATGC-3′). The PCR reaction mix for both bacterial 16S and fungal ITS region amplifications was a 50 μL system, consisting of 25 μL EXtaq enzyme (TaKaRa, Kyoto, Japan), 21 μL ddH_2_O, 1 μL of the forward primer, 1 μL of the reverse primer, and 2 μL of DNA template. The amplification conditions for the bacterial 16S rRNA gene region were as follows: initial denaturation at 94 °C for 3 min, followed by 35 cycles of denaturation at 94 °C for 45 s, annealing at 50 °C for 1 min, and extension at 72 °C for 1 min and 30 s. A final extension was performed at 72 °C for 10 min, followed by a hold at 4 °C. The amplification conditions for the fungal ITS region were as follows: initial denaturation at 94 °C for 1 min, followed by 35 cycles of denaturation at 94 °C for 30 s, annealing at 52 °C for 30 s, and extension at 72 °C for 30 s. A final extension was performed at 72 °C for 10 min, followed by a hold at 4 °C.

The PCR amplification products were purified using the Gel Extraction Kit (Omega Bio-Tek, Norcross, GA, USA) according to the manufacturer’s instructions. The purified amplicons were sequenced using the HiSeq 2500 platform (Illumina, Inc., San Diego, CA, USA). All raw sequence data were deposited in NCBI Sequence Read Archive (SRA) database under accession number PRJNA1213772.

### 4.6. Data Analyses

We employed linear mixed-effects models to assess (1) the effect of relatedness on the focal cultivar’s grain yield and flowering time in the 2021 field trials; (2) the effects of cultivars and their interaction on neighbor cultivars’ grain yield and flowering time in the 2021 field trials; (3) the effects of relatedness, years, and planting patterns and their interactions on grain yield, flowering time, and root-to-shoot ratio at both the seedling and mature stages in the 2022 and 2023 field trials. The models incorporated plot (nested within block) as a random effect to account for the same plot being measured repeatedly over time. For pot experiments, linear models were used to test for the effects of relatedness, soil volume, and their interaction on root-to-shoot ratio, as well as the effects of relatedness, nitrogen levels, and their interaction on the grain yield and root-to-shoot ratio. Post hoc comparisons among relatedness levels were conducted using Tukey’s HSD test at a significance level of *p* = 0.05. All fitted models were performed in R v.4.4.2 (http://www.r-project.org, accessed on 15 June 2025) using the lme4 package [57]. Residuals were checked for normality and homoscedasticity, and log_e_-transformation was applied to variables when necessary to meet model assumptions.

Raw reads of sequencing were processed and applied to the DADA2 pipeline for the assignment of amplicon sequence variants (ASVs) [58]. The DADA2 workflow includes filtering, dereplication, chimera identification, and clustering into ASVs. Taxonomy was assigned to the resulting unique ASVs using the SILVA database [59] for bacterial 16S rRNA gene sequences and the UNITE database [60] for fungal ITS sequences. Before performing the diversity analysis, the sequences were rarefied to a minimum sequencing depth of 20,000 reads. α-diversity indices (observed species richness, Chao 1, and Shannon index) were calculated in R using the “vegan” package. β-diversity was assessed using a Bray–Curtis distance matrix, and principal coordinate analyses (PCoA) was conducted to determine the similarity among microbial communities in different samples based on this dissimilarity, using the “vegan” package. To determine whether there were significant differences among different groups based on the Bray–Curtis dissimilarity matrix, permutational multivariate analysis of variance (PERMANOVA) was conducted using the adonis function in the vegan package [61]. Relative abundance was estimated on the phylum and genus level. The heat map of top 30 relative abundance genera was plotted using the R-package “ComplexHeatmap” [62].

## 5. Conclusions

Cultivar mixtures have been shown to increase both crop yield and temporal stability globally, benefitting agriculture. However, most successful mixtures have been established from traditional practices or are assessed from haphazardly built experimental combinations. Currently, there is still a lack of effective strategies for the a priori selection of mixture components to achieve agricultural benefits. This study provides compelling evidence that neighbor relatedness can contribute to yield improvements in combinations of two rice cultivars. Although the effect of relatedness is the consequence of complex interactions among rice cultivars, the microbiome, and soil nutrient cycling and utilization processes, cultivar kinship plays a crucial role in determining the outcome of intraspecific plant–plant interactions in cultivar mixtures. The question of how to identify suitable closely related pairings from cultivars with varying degrees of genetic relatedness needs to be refined, but it is warranted to consider neighbor relatedness in designing beneficial cultivar mixtures. Future research should expand to larger-scale field trials with quantitative genetic distance metrics to precisely map how kinship effects scale with genetic divergence. The predictive potential of kinship strategies in cultivar mixtures is very intriguing. A further understanding of neighbor relatedness for improving performance and yield in cultivar mixtures may allow for large-scale implications and applications to increase yields in the limited area suitable for agriculture.

## Data Availability

All data supporting the findings of this paper are available within the paper and its Supplementary Materials published online. The raw 16S rRNA amplicon-seq and RNA-seq reads reported in this paper have been deposited to National Center for Biotechnology Information (NCBI Short Read Archive under Project PRJNA1205921).

## Supplementary Materials

The following supporting information can be downloaded at https://www.mdpi.com/article/10.3390/plants14152385/s1: Figure S1. Root-to-shoot ratio of focal cultivars (Huagan-3) in monoculture and mixtures of closely and distantly related cultivars at seedling and mature stage across different planting patterns (cross vs. row) in two years (2022 vs. 2023). Figure S2. The average temperature and precipitation during the field trials in 2022 and 2023. Figure S3. Pedigree and genetic distance of rice cultivars used in this study. Figure S4. Field site and experiment design. Figure S5. The illustration of the experimental design for the greenhouse experiments. Table S1. Results of linear mixed-effect models for the field trials in different plant patterns and years. Table S2. Analysis of variance for volume-mediated experiments. Table S3. Analysis of variance for the nitrogen gradient experiments. Table S4. The alpha diversity (observed species richness, Chao1, and Shannon indices) of bacteria and fungi in rhizosphere soil incubated with focal rice cultivars mixed with different related cultivars at seedling stage.
