# Supplementary material for: Neighbor Relatedness Contributes to Improvement in Grain Yields in Rice Cultivar Mixtures
Source: Plants (Basel). 2025 Aug 2;14(15):2385. doi: 10.3390/plants14152385 (PMC12349112; doi:10.3390/plants14152385)
Supplement: Supplementary file 1 [file plants-14-02385-s001.zip › plants-3732392-supplementary.pdf]

plants

MDPI

Supplementary materials

Neighbor relatedness contributes to improvement of grain yields in rice cultivar mixtures

You Xu [1,2], Qin-Hang Han [1], Shuai-Shuai Xie [1] and Chui-Hua Kong [1,*]

This supplementary material was prepared to add the readers more details, for which there was not enough space in the main manuscript, about:

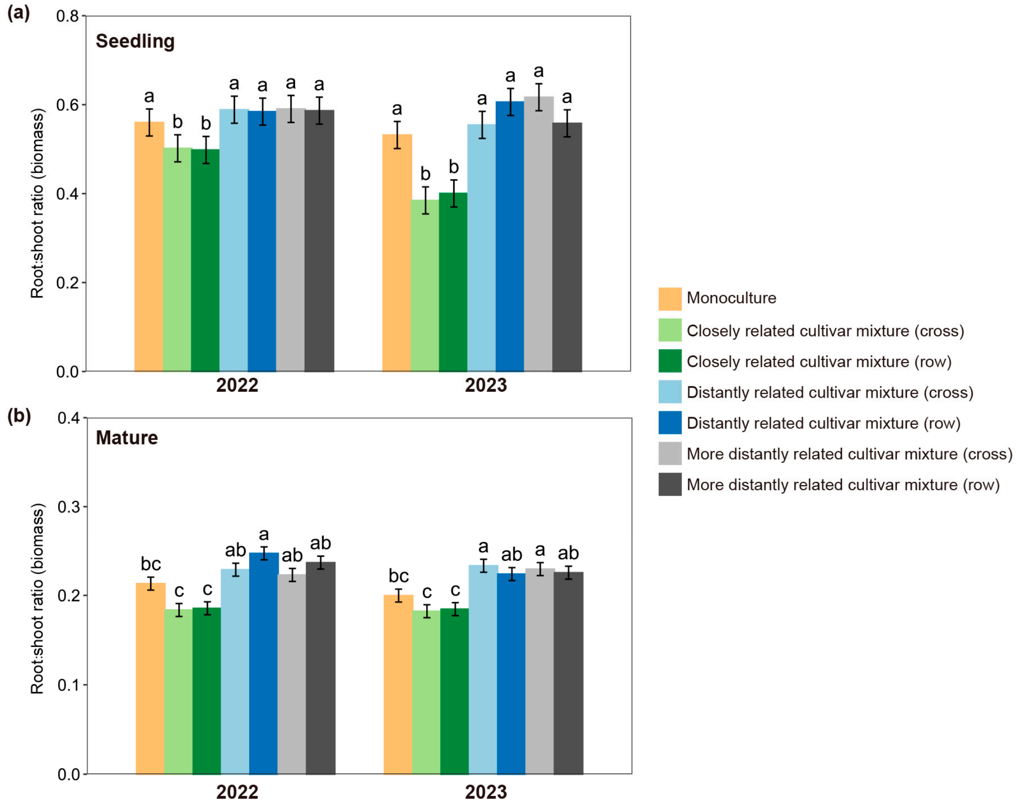

Figure S1. Root-to-shoot ratio of focal cultivars (Huagan-3) in monoculture and mixtures of closely and distantly related cultivars at seedling and mature stage across different planting pattern (cross vs. row) in two years (2022 vs. 2023). Closely related cultivar, Huagan-8; Distantly related cultivar, Huafeng (indica); More distantly related cultivar, Liaojing-9 (japonica). Values plotted are means ± SE. Columns with the same letter are not significantly different among relatedness and planting pattern at $P < 0.05$ followed by Tukey's HSD tests.

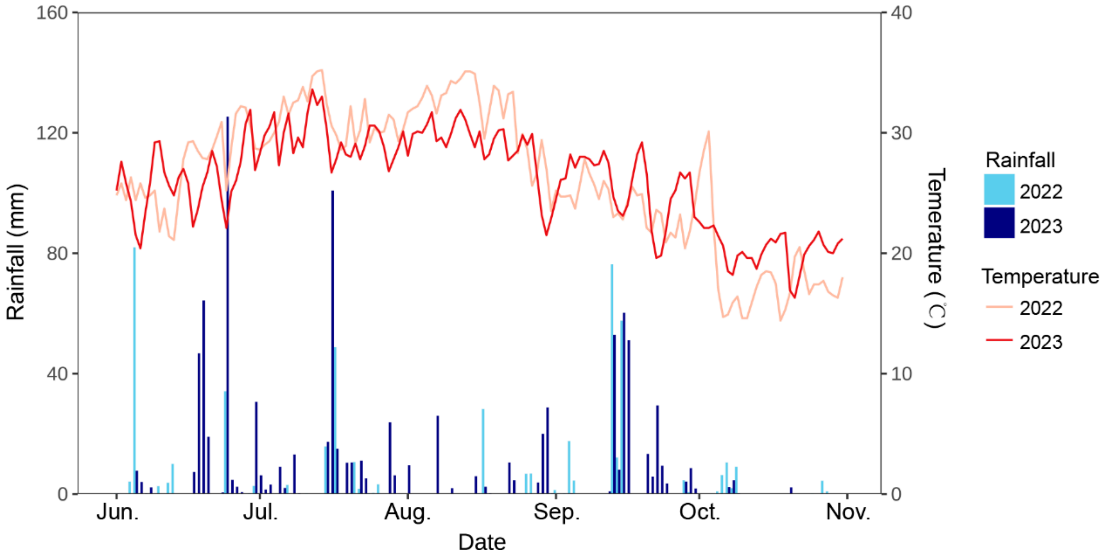

Figure S2. The average temperature and precipitation during the field trials in 2022 and 2023. The fields located at Suzhou Rice Experimental Station of China Agricultural University, Jiangsu Province, China.

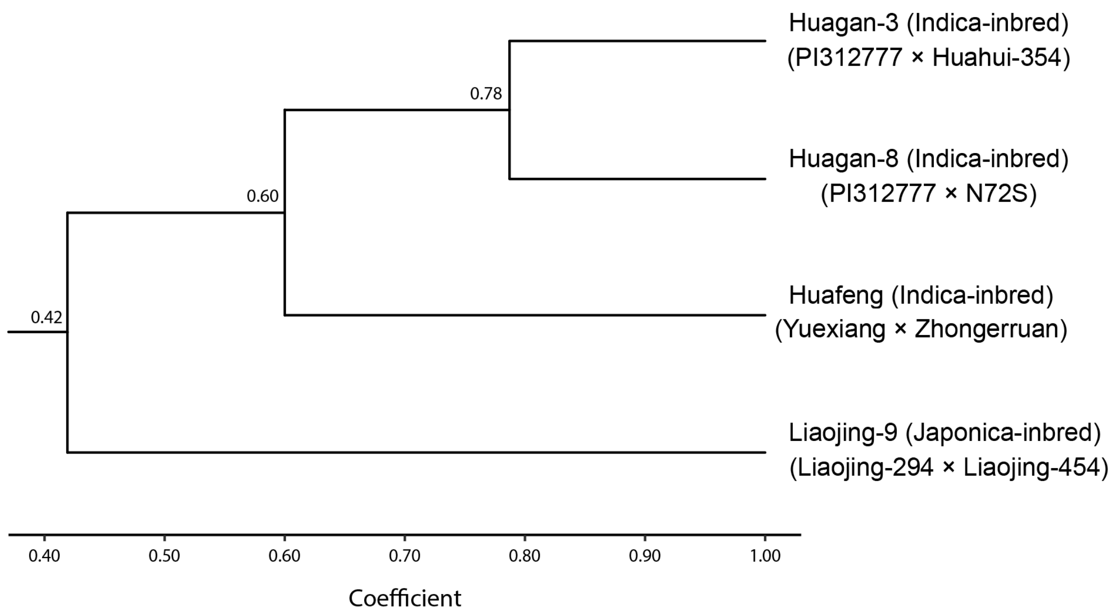

Figure S3. Pedigree and genetic distance of rice cultivars used in this study. Genetic distances of the rice lines relative to the focal cultivar (Huagan-3) were analyzed using simple sequence repeat markers.

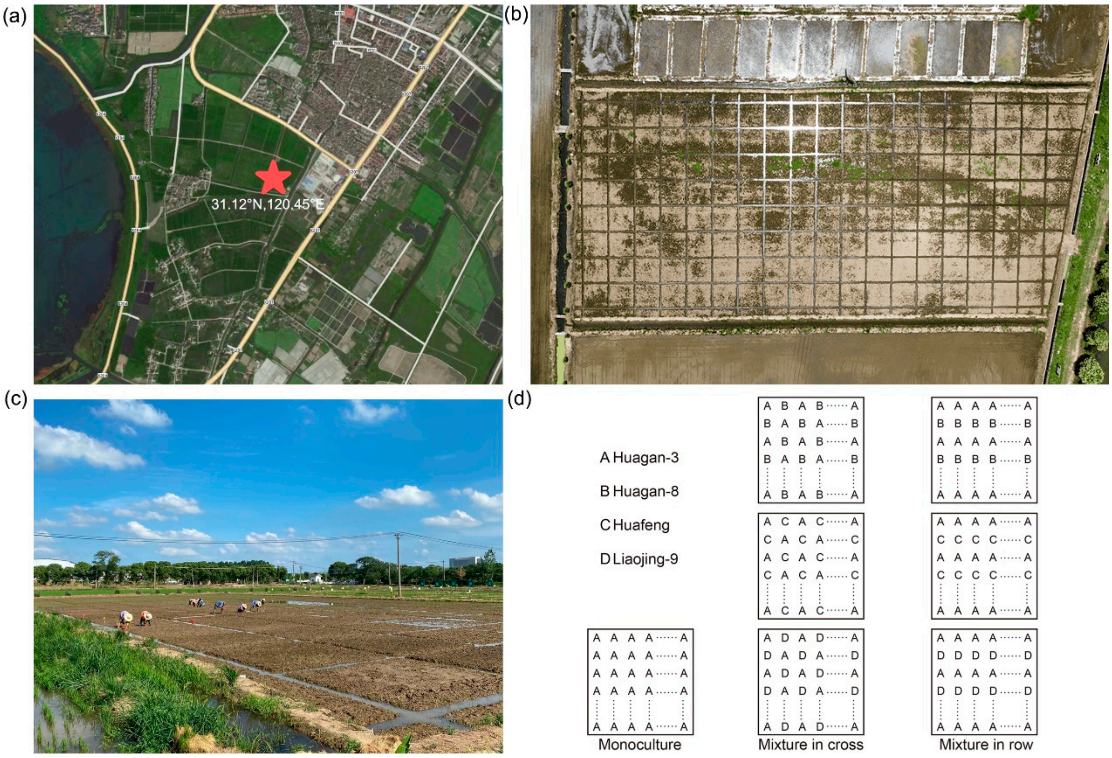

Figure S4. Field site and experiment design. **(a)** Satellite image of field site at Suzhou Rice Experimental Station of China Agricultural University, Jiangsu Province, China. **(b)** Aerial image of the field experiment plots. **(c)** Image of field experiment in sowing stage. **(d)** Experimental design diagram.

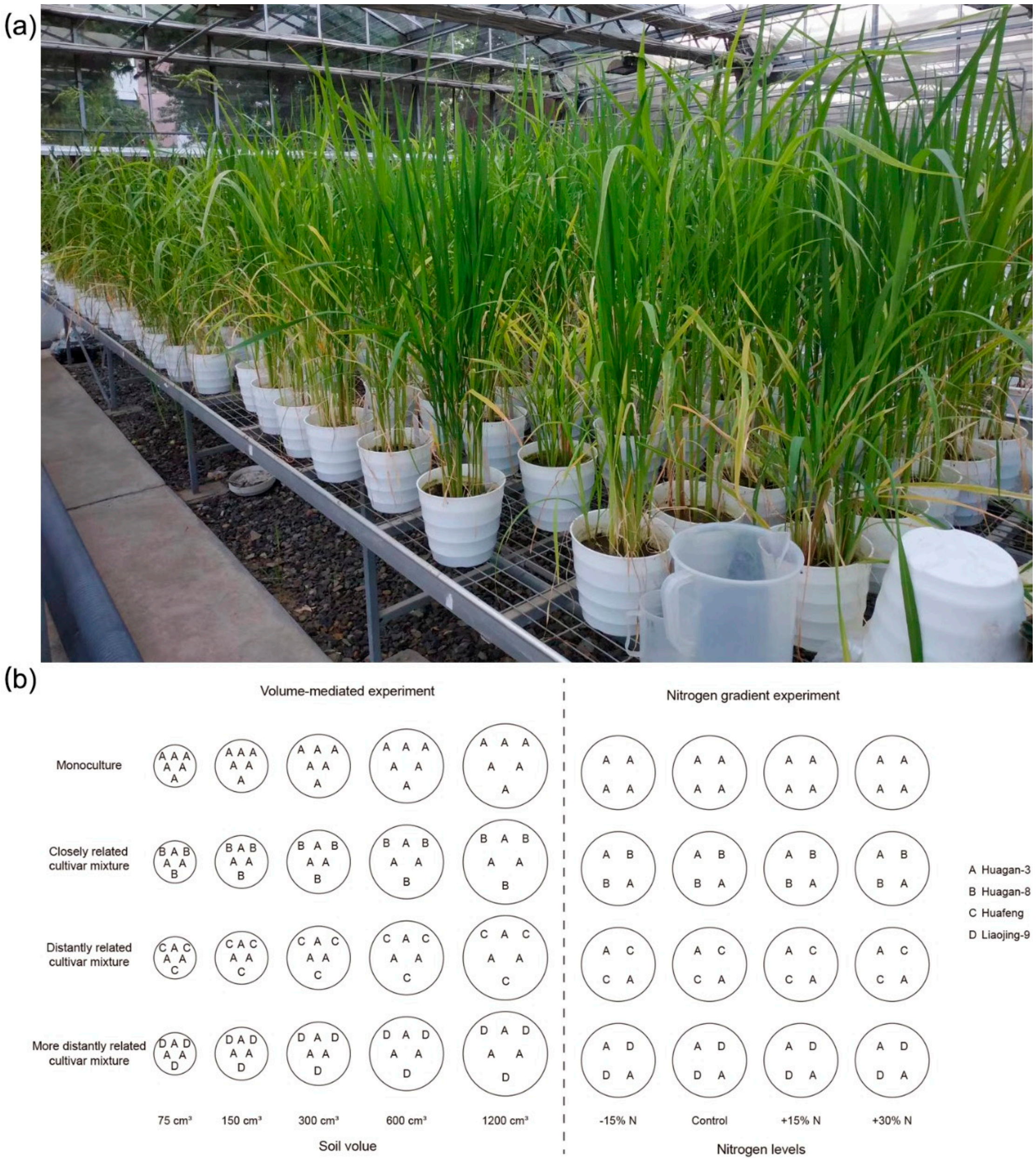

Figure S5. The illustration of the experimental design for the greenhouse experiments. **(a)** Image of pot experiment in greenhouse. **(b)** Diagram of volume-mediated and nitrogen gradient experiments.

Table S1. Results of linear mixed-effect models for the field trials in different plant pattern and years. Effects are relatedness (Monoculture, closely related cultivar, distantly related cultivar and more distantly related cultivar), year (2022 vs. 2023) and plant pattern (cross vs. row). Significance of effects was determined by Satterthwaite' s approximate F-test.

Effects	NumDF	Grain yield			Flowering time			Root-shoots ratio (seedling)			Root-shoots ratio (mature)		
		DenDF	*F*	*P*	DenDF	*F*	*P*	DenDF	*F*	*P*	DenDF	*F*	*P*
Relatedness (R)	3	12.730	10.146	0.001	34.526	33.293	<0.001	11.804	17.070	<0.001	15.944	23.740	<0.001
Years (Y)	1	27.269	27.133	<0.001	6.108	0.096	0.767	28.800	5.421	0.027	29.321	4.269	0.048
Plant pattern (PP)	1	22.119	0.972	0.335	45.378	0.024	0.879	30.462	0.001	0.979	25.771	0.752	0.394
R×Y	3	27.269	0.202	0.894	34.526	0.911	0.446	28.800	2.576	0.073	29.321	0.903	0.452
R×PP	3	22.678	0.221	0.881	49.442	0.523	0.669	27.070	0.550	0.653	22.767	0.061	0.980
Y×PP	1	27.269	0.304	0.586	45.378	0.016	0.901	28.800	0.028	0.869	29.321	3.190	0.084
R×Y×PP	3	27.269	0.180	0.909	49.442	0.056	0.983	28.800	0.565	0.643	29.321	0.983	0.414
(random factor) Block/Plot													

Table S2. Analysis of variance for volume-mediated experiments. Effects are relatedness (Monoculture, closely related cultivar mixture, distantly related cultivar mixture and more distantly related cultivar mixture), soil volume (75 cm^3, 150 cm^3, 300 cm^3, 600 cm^3 and 1200 cm^3) and their interaction.

**Effects**	**Root- shoots ratio**		
	**DF**	***F***	***P***
Relatedness (R)	3	25.736	<0.001
Soil volume (SV)	4	17.294	<0.001
R×SV	12	0.180	0.0178
Residuals	60		

Table S3. Analysis of variance for the nitrogen gradient experiments. Effects are Effects are relatedness (Monoculture, closely related cultivar mixture, distantly related cultivar mixture and more distantly related cultivar mixture), nitrogen levels (-15% N, Control, +15% N and +30% N) and their interaction.

**Effects**	**DF**	**Grain yield**		**Root- shoots ratio**	
		F	***P***	***F***	***P***
Relatedness (R)	3	43.847	<0.001	22.385	<0.001
Nitrogen levels (NL)	3	23.357	<0.001	11.521	<0.001
R×NL	9	3.431	<0.001	0.578	0.813
Residuals	112				

Table S4. The alpha diversity (observed species richness, Chao1 and Shannon indices) of bacteria and fungi in rhizosphere soil incubated with focal rice cultivars mixed with different related cultivars at seedling stage.

Microbes	Relatedness	Richness	Chao1 index	Shannon index
Bacteria	Monoculture	2447.50±44.19 b	3967.66±234.28 a	6.81±0.01 bc
	Closely related cultivar mixture	2591.25±86.51 a	4243.06±265.42 a	6.94±0.04 a
	Distantly related cultivar mixture	2396.25±52.43 b	4022.67±160.87 a	6.73±0.05 c
	More distantly related cultivar mixture	2433.75±75.71 b	4136.24±96.81 a	6.83±0.06 b
Fungi	Monoculture	193.50±5.57 b	250.60±5.58 a	2.55±0.17 c
	Closely related cultivar mixture	234.50± 11.09 a	275.34±29.40 a	3.52±0.20 a
	Distantly related cultivar mixture	203.50±7.59 b	259.50±27.15 a	3.00±0.09 b
	More distantly related cultivar mixture	204.00±11.20 b	279.07±63.04 a	3.12±0.06 b
